# Supplementary material for: Transcriptome Analysis of the Oriental Fruit Fly Bactrocera dorsalis Early Embryos
Source: Insects. 2020 May 23;11(5):323. doi: 10.3390/insects11050323 (PMC7290859; doi:10.3390/insects11050323)
Supplement: Supplementary file 1 [file insects-11-00323-s001.zip › Supplementary Material/Table S1.docx]

**Table S5** Primers used in our study

| Primer names | Sequence 5’ to 3’ |
| --- | --- |
| *Bdtra* F | TTCCAAAACTGTTGGCAACATCAAG |
| *Bdtra* R | GAACCTTCACCGAATCTACG |
| *Bddsx* F | ACCCTGTAGATCCGAATTT |
| *Bddsx* R | CAAATTCGGTTCTAGAGAGG |
| *Bdtra-2* F | TGAGTATTACATCAGGTAC |
| *Bdtra-2* R | TAGGAACTTCATACCGTGCTCT |
| *Bdfru* F | CACTACAAGCCTCACGAA |
| *Bdfru* R | GTGGGTAGGAGAAAGCAG |
| *BdSxl* F | TGTACGCTTAGCCGAGGAG |
| *BdSxl* R | GCATCTTTTGGAAACGTGAT |
| *Bdda* F | TCAAATCCTTCAACGCCAGTA |
| *Bdda R* | TTGAGAACCGCTCCCCAT |
| *Bdfl(2)d* F | GGCGGCAATGGTTTAGGT |
| *Bdfl(2)d* R | GCGATGGTGGCTGATGTT |
| *Bdotu* F | CTGAAGAGTTGGTGGGTA |
| *Bdotu* R | GCATTGCAGGATCATAAC |
| *Bddpn* F | CGCCCGTCATACCAAACT |
| *Bddpn* R | CTTCCTCGGCACATTCTA |
| *Rp49* F | CCCGTCATATGCTGCCAACT |
| *Rp49* R | GCGCGCTCAACAATTTCCTT |
