# Supplementary figures and images for: Transcriptome Analysis of the Oriental Fruit Fly Bactrocera dorsalis Early Embryos
Source: Insects. 2020 May 23;11(5):323. doi: 10.3390/insects11050323 (PMC7290859; doi:10.3390/insects11050323)

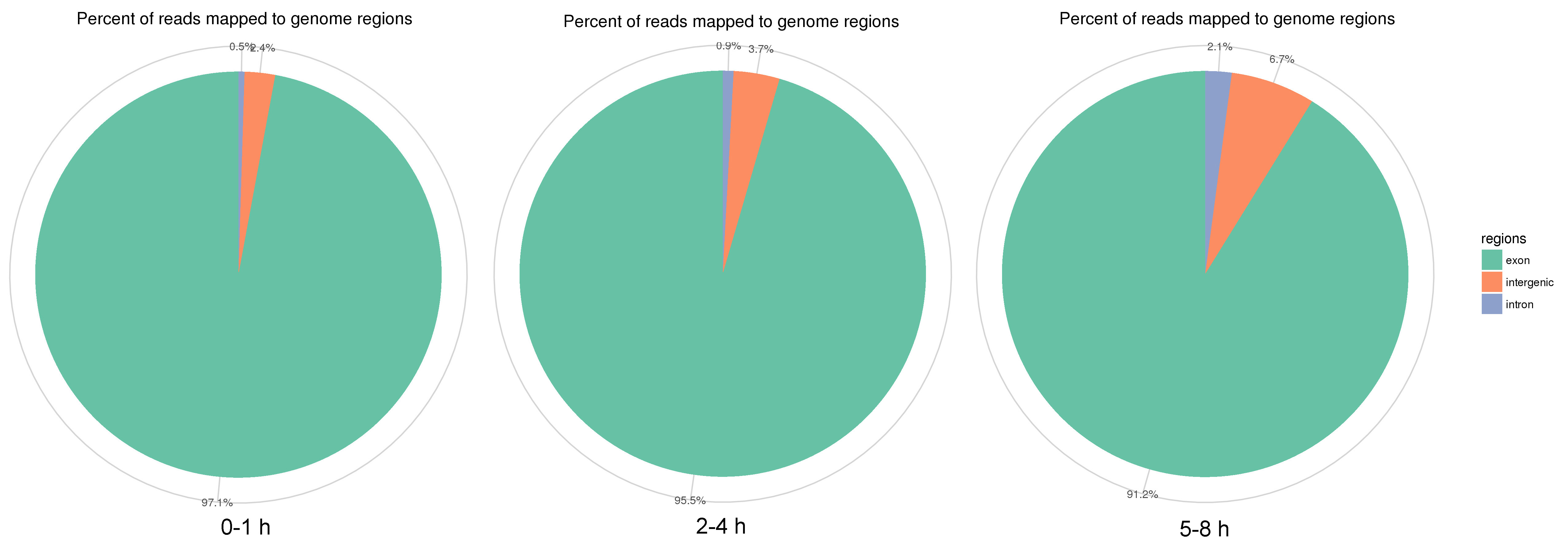

Supplement: Supplementary file 1 [file insects-11-00323-s001.zip › Supplementary Material/Figure S3.jpg]

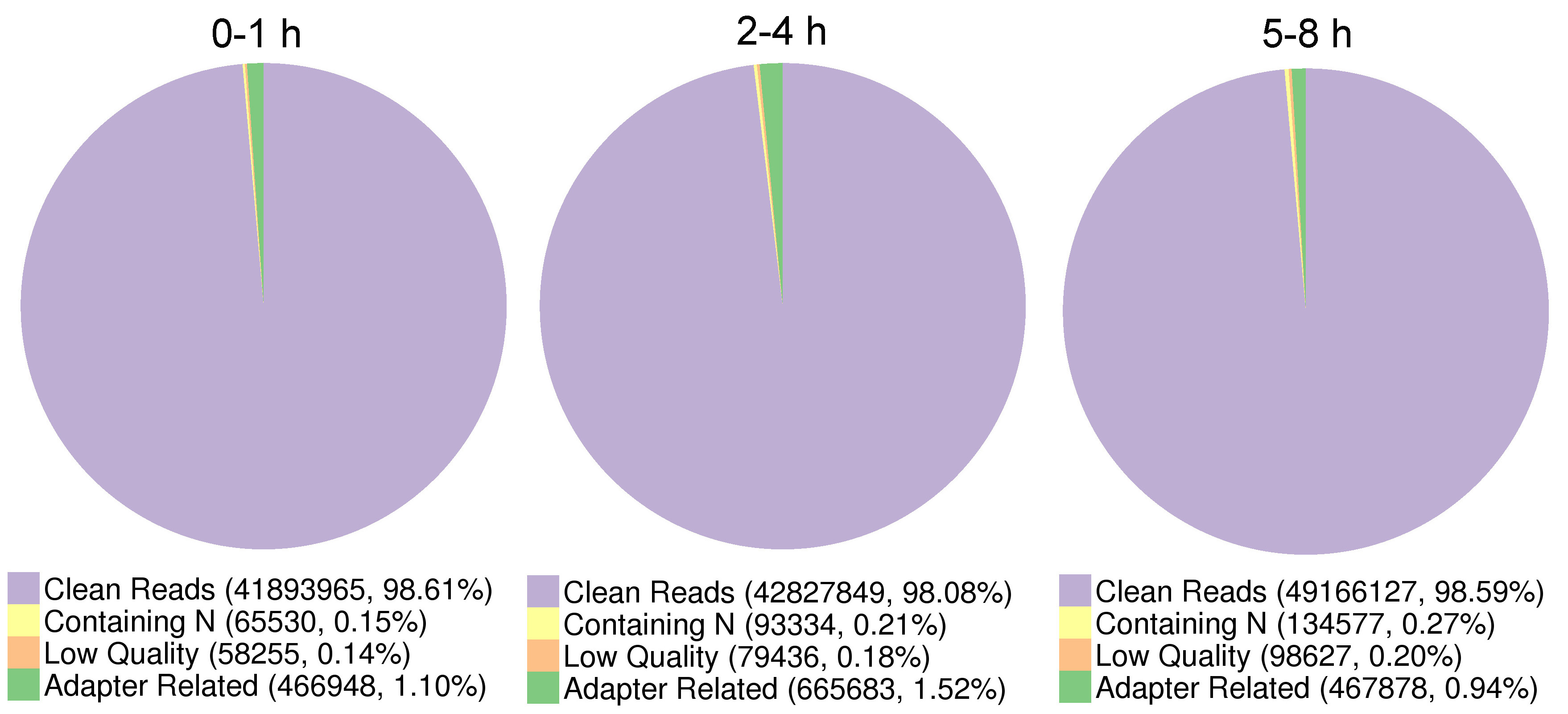

Supplement: Supplementary file 1 [file insects-11-00323-s001.zip › Supplementary Material/Figure S2.jpg]

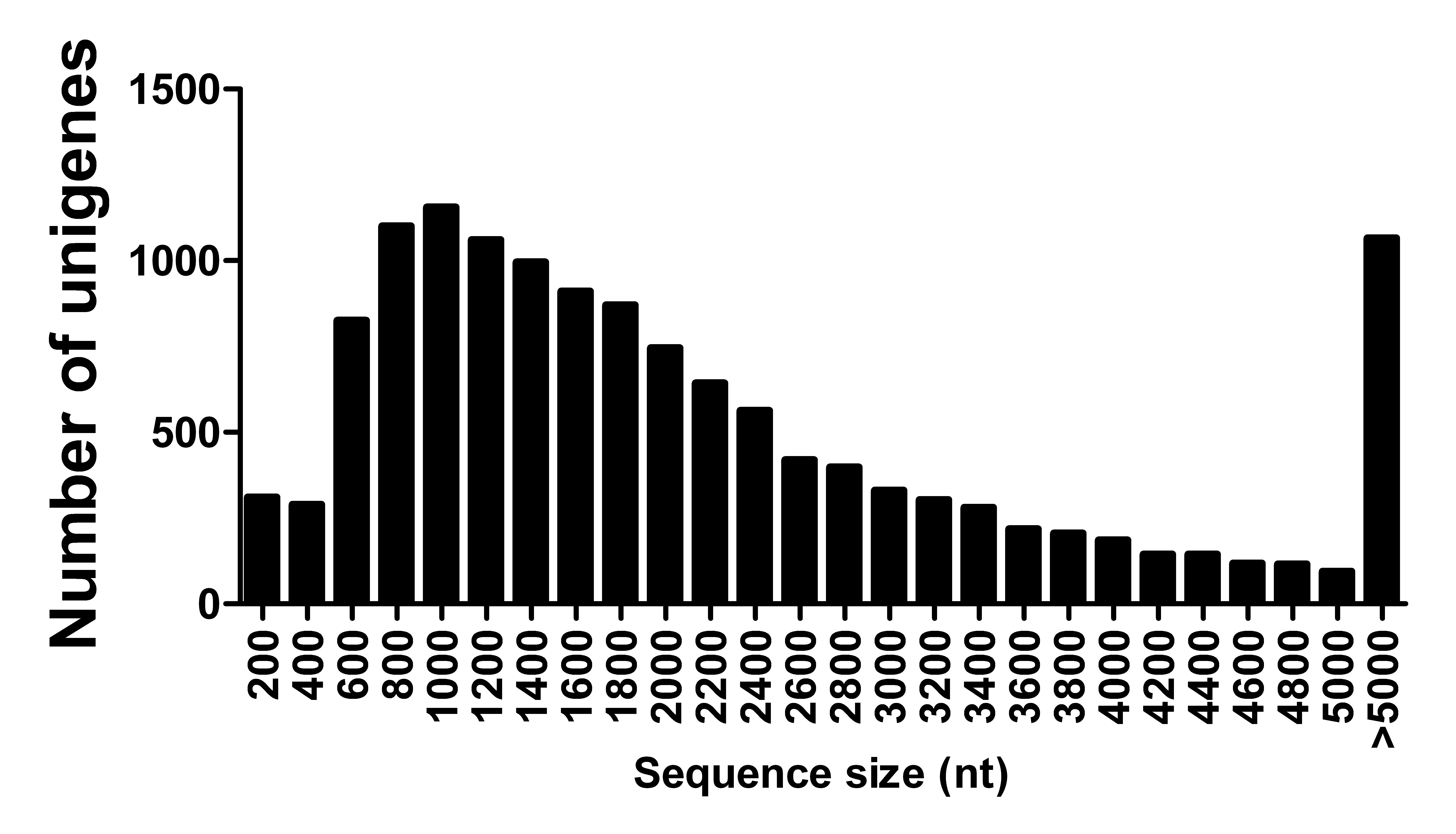

Supplement: Supplementary file 1 [file insects-11-00323-s001.zip › Supplementary Material/Figure S1.jpg]

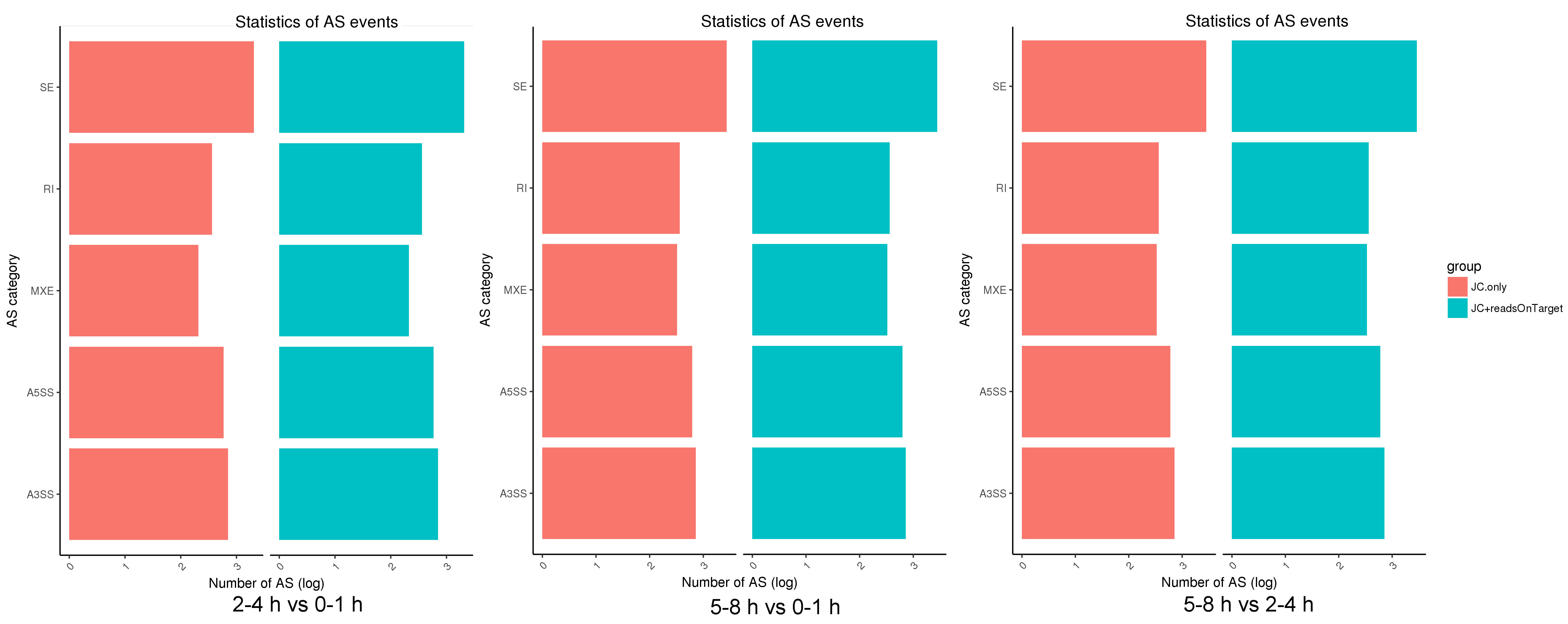

Supplement: Supplementary file 1 [file insects-11-00323-s001.zip › Supplementary Material/Figure S4.jpg]
